# Book Review

**DOI:** 10.1120/jacmp.v2i4.2600

**Published:** 2001-09-01

**Authors:** Marilyn Stovall

**Affiliations:** ^1^ Radiation Physics Department 1515 Holcombe Blvd., Box 544 Houston TX 77030

## Abstract

**Liver Cancer Risk from Internally‐Deposited Radionuclides.** NCRP Report No. 135., 94 pp. National Council on Radiation Protection and Measurement, Bethesda, MD, 2001. ISBN: 0‐929600‐68‐1. Price: $20.00.

PACS number(s): 87.52.Px, 87.58.Ji

NCRP Report No. 135 provides the latest estimates of liver cancer risk from radionuclides deposited in the liver and updates data published by the Committee on Biological Effects of Ionizing Radiation (BEIR, 1988) and the United Nations Scientific Committee on the Effects of Atomic Radiation (UNSCEAR, 1994). In general, the report raises previous risk estimates and refutes the view that the liver is a radioresistant organ.

Estimates of cancer risk from *α* and *β‐γ* emitters were derived using numerous extrapolations from human experience with Thorotrast® and molecular, cellular, and animal experiments. The extensive publications cited by the authors serve to strengthen their conclusions.

In addition to absorbed dose and dose distribution, there is discussion of other considerations that impact the evaluation of cancer risk: latent period, biological half‐life, and “wasted dose,” as well as the bystander effect at the cellular level.

Of necessity, there are large uncertainties associated with risk estimates in humans due to the numerous extrapolations. Furthermore, assessment of risk in an individual is complicated by the influence of various host factors (age, sex, other disease states, genetic background) and exposure to environmental carcinogens. The discussion of uncertainties underscores the fact that we are far from unraveling all the mysteries of this complex organ.

As one would expect from the NCRP, the report is concise, clear, and well‐organized. It is an essential reference for anyone working in nuclear medicine or radiation protection.

